# Supplementary material for: Humanized single domain antibodies neutralize SARS-CoV-2 by targeting the spike receptor binding domain
Source: Nat Commun. 2020 Sep 10;11:4528. doi: 10.1038/s41467-020-18387-8 (PMC7483421; doi:10.1038/s41467-020-18387-8)
Supplement: Supplementary file 1 — Supplementary information [file 41467_2020_18387_MOESM1_ESM.pdf]

Supplemental information

Humanized Single Domain Antibodies Neutralize SARS-CoV-2 by Targeting the Spike Receptor Binding Domain

Chi et al.

Supplemental Table 1. Phage ELISA reactivity at OD450 of individual sdAb clone towards SARS-CoV-2 RBD, mouse IgG1 Fc and SARS-CoV RBD.

PLATE #1

SARS-COV-2 RBD, OD450

	1	2	3	4	5	6	7	8	9	10	11	12
**A**	0.431	0.779	0.577	0.449	0.262	0.11	0.069	0.115	0.29	0.811	0.229	0.809
**B**	1.038	0.798	0.498	0.638	0.948	0.654	0.13	0.821	0.168	0.052	0.335	0.122
**C**	0.571	0.609	0.304	0.216	0.373	0.33	0.335	0.535	0.349	0.353	0.131	0.05
**D**	0.065	0.055	0.061	0.051	0.053	0.054	0.067	0.054	0.053	0.049	0.051	0.052
**E**	1.665	**0.886**	0.673	0.754	0.65	0.622	0.31	0.5	0.055	0.156	0.134	0.285
**F**	0.456	0.482	0.458	0.725	0.304	0.12	0.486	0.086	0.439	0.366	0.349	0.515
**G**	1.599	0.333	0.049	0.571	0.697	0.729	0.094	0.131	0.603	0.25	0.423	0.488
**H**	0.073	0.093	0.322	0.43	1.148	0.058	0.478	0.591	0.849	0.416	0.501	0.087

mouse Fc, OD450

	1	2	3	4	5	6	7	8	9	10	11	12
**A**	0.051	0.053	0.053	0.051	0.059	0.053	0.056	0.055	0.054	0.055	0.053	0.057
**B**	0.055	0.055	0.052	0.062	0.062	0.067	0.079	0.084	0.072	0.083	0.057	0.069
**C**	0.057	0.054	0.055	0.053	0.056	0.057	0.053	0.053	0.051	0.055	0.051	0.055
**D**	0.057	0.066	0.059	0.06	0.063	0.062	0.062	0.053	0.063	0.063	0.059	0.058
**E**	0.133	0.059	0.057	0.065	0.06	0.064	0.068	0.058	0.062	0.058	0.054	0.058
**F**	0.076	0.065	0.064	0.066	0.062	0.2	0.066	0.062	0.066	0.065	0.062	0.059
**G**	0.053	0.086	0.054	0.057	0.057	0.054	0.066	0.063	0.057	0.057	0.057	0.05
**H**	0.049	0.064	0.147	0.058	0.067	0.066	0.054	0.05	0.05	0.049	0.047	0.045

SARS-COV RBD, OD450

	1	2	3	4	5	6	7	8	9	10	11	12
**A**	0.081	0.048	0.049	0.047	0.054	0.049	0.054	0.054	0.061	0.059	0.048	0.048
**B**	0.051	0.054	0.052	0.051	0.051	0.052	0.048	0.08	0.051	0.066	0.051	0.052
**C**	0.046	0.047	0.048	0.049	0.047	0.047	0.049	0.053	0.051	0.061	0.047	0.048
**D**	0.05	0.051	0.053	0.05	0.052	0.051	0.048	0.066	0.051	0.054	0.052	0.052
**E**	0.457	0.05	0.051	0.248	0.052	0.048	0.058	0.051	0.05	0.049	0.048	0.047
**F**	0.053	0.056	0.095	0.065	0.053	0.415	0.087	0.058	0.051	0.052	0.052	0.053
**G**	0.049	0.05	0.05	0.05	0.059	0.049	0.049	0.05	0.05	0.054	0.051	0.049
**H**	0.041	0.046	0.915	0.045	0.044	0.069	0.046	0.049	0.348	0.048	0.05	0.045

PLATE #2

SARS-COV-2 RBD, OD450

	1	2	3	4	5	6	7	8	9	10	11	12
**A**	0.391	0.965	0.849	0.162	0.866	0.165	0.11	1.441	0.343	1.571	0.345	1.386
**B**	1.715	1.346	1.984	0.324	0.659	0.456	0.464	0.057	1.32	0.627	0.222	1.278
**C**	0.251	0.224	0.481	0.089	0.321	0.136	0.113	0.679	0.918	0.512	1.96	0.06
**D**	0.255	0.445	0.748	0.105	0.432	0.425	0.107	0.64	0.964	0.05	0.049	0.059
**E**	0.745	0.398	0.993	0.212	0.536	1.165	1.497	0.317	0.524	0.691	0.307	0.045
**F**	1.811	**1.042**	0.89	0.807	0.893	0.784	1.776	1.223	1.219	0.664	0.156	0.057
**G**	0.288	0.194	0.942	0.673	0.735	1.336	0.551	0.051	0.435	2.362	0.78	0.653
**H**	0.641	0.248	0.869	0.651	0.416	1.071	1.278	1.189	1.596	1.446	1.636	0.145

mouse Fc, OD450

	1	2	3	4	5	6	7	8	9	10	11	12
**A**	0.049	0.049	0.049	0.08	0.072	0.049	0.049	0.056	0.053	0.054	0.047	0.051
**B**	0.05	0.057	0.067	0.057	0.059	0.075	0.063	0.061	0.057	0.056	0.053	0.053
**C**	0.049	0.052	0.071	0.098	0.054	0.052	0.057	0.059	0.081	0.078	0.052	0.052
**D**	0.054	0.054	0.056	0.076	0.057	0.071	0.056	0.068	0.057	0.056	0.051	0.052
**E**	0.051	0.053	0.063	0.063	0.057	0.061	0.056	0.06	0.053	0.055	0.051	0.048
**F**	0.067	0.059	0.054	0.077	0.057	0.06	0.067	0.066	0.062	0.053	0.05	0.051
**G**	0.061	0.057	0.057	0.07	0.074	0.051	0.059	0.064	0.052	0.048	0.048	0.049
**H**	0.049	0.049	0.054	0.065	0.055	0.049	0.05	0.07	0.056	0.047	0.047	0.044

SARS-COV RBD, OD450

	1	2	3	4	5	6	7	8	9	10	11	12
**A**	0.047	0.051	0.048	0.14	0.071	0.046	0.045	0.046	0.046	0.046	0.046	0.044
**B**	0.048	0.048	0.163	0.052	0.052	0.056	0.06	0.053	0.049	0.061	0.047	0.061
**C**	0.048	0.047	0.049	0.098	0.045	0.044	0.045	0.048	0.046	0.046	0.045	0.046
**D**	0.05	0.058	0.276	0.048	0.053	0.047	0.048	0.049	0.051	0.048	0.049	0.062
**E**	0.049	0.05	0.048	0.049	0.049	0.046	0.048	0.048	0.048	0.046	0.045	0.048
**F**	0.447	0.052	0.05	0.053	0.051	0.053	0.054	0.054	0.058	0.064	0.054	0.058
**G**	0.048	0.303	0.048	0.047	0.047	0.05	0.05	0.048	0.048	0.329	0.053	0.048
**H**	0.044	0.045	0.044	0.044	0.042	0.042	0.042	0.043	0.042	0.042	0.042	0.042

-continued

PLATE #3

SARS-COV-2 RBD, OD450

	1	2	3	4	5	6	7	8	9	10	11	12
**A**	0.675	0.478	1.812	0.279	1.151	0.807	0.054	1.158	0.952	0.046	0.095	0.047
**B**	0.266	1.153	0.975	0.417	0.503	0.059	1.533	0.172	0.096	0.798	0.888	0.373
**C**	0.576	0.228	0.894	0.046	0.078	0.139	0.104	0.21	0.088	1.112	1.359	1.671
**D**	0.198	0.646	1.31	1.429	0.766	0.255	0.586	0.591	0.341	0.117	0.294	0.463
**E**	1.376	0.191	1.48	0.071	0.13	0.045	0.064	0.066	0.303	0.059	0.167	0.303
**F**	1.26	0.052	1.3	1.661	0.466	0.644	0.117	1.004	0.502	0.245	**2.125**	0.295
**G**	0.999	1.009	0.85	0.135	0.903	0.129	1.381	0.145	0.047	0.11	0.843	0.755
**H**	0.213	1.68	1.052	1.178	0.614	0.093	0.146	0.107	0.079	0.083	0.042	0.042

mouse Fc, OD450

	1	2	3	4	5	6	7	8	9	10	11	12
**A**	0.05	0.052	0.05	0.05	0.052	0.054	0.049	0.051	0.057	0.055	0.049	0.049
**B**	0.057	0.054	0.053	0.051	0.056	0.052	0.078	0.062	0.061	0.08	0.073	0.088
**C**	0.051	0.047	0.046	0.05	0.047	0.05	0.049	0.09	0.062	0.052	0.049	0.048
**D**	0.056	0.054	0.053	0.051	0.06	0.056	0.055	0.056	0.058	0.06	0.057	0.059
**E**	0.055	0.049	0.067	0.049	0.048	0.05	0.05	0.047	0.05	0.048	0.048	0.048
**F**	0.056	0.053	0.052	0.054	0.053	0.051	0.056	0.055	0.083	0.082	0.056	0.063
**G**	0.05	0.05	0.053	0.05	0.049	0.049	0.048	0.049	0.056	0.05	0.05	0.051
**H**	0.043	0.047	0.045	0.046	0.046	0.045	0.053	0.042	0.06	0.046	0.047	0.043

SARS-COV RBD, OD450

	1	2	3	4	5	6	7	8	9	10	11	12
**A**	0.083	0.076	0.084	0.080	0.414	0.079	0.080	0.081	0.278	0.083	0.082	0.083
**B**	0.096	0.081	0.077	0.080	0.075	0.075	0.631	0.080	0.076	0.181	0.089	0.089
**C**	0.080	0.079	0.081	0.085	0.081	0.077	0.071	0.071	0.073	0.079	0.093	0.086
**D**	0.084	0.082	0.078	0.078	0.080	0.077	0.208	0.072	0.120	0.071	0.135	0.153
**E**	0.090	0.082	0.084	0.077	0.077	0.079	0.073	0.073	0.075	0.071	0.084	0.150
**F**	0.084	0.076	0.080	0.081	0.078	0.076	0.091	0.083	0.152	0.127	0.151	0.121
**G**	0.597	0.081	0.075	0.085	0.076	0.078	0.084	0.085	0.082	0.126	0.295	0.268
**H**	0.090	0.831	0.905	0.795	0.087	0.101	0.131	0.121	0.122	0.183	0.245	0.238

-continued

PLATE #4

SARS-COV-2 RBD, OD450

	1	2	3	4	5	6	7	8	9	10	11	12
**A**	0.255	0.753	0.743	0.103	0.729	0.325	0.708	0.857	0.751	0.156	1.131	0.154
**B**	0.891	0.583	0.645	0.151	1.464	0.047	1.097	1.132	0.986	1.228	1.364	0.534
**C**	0.717	0.613	0.702	0.937	0.052	0.902	1.338	1.351	0.904	0.979	0.458	0.97
**D**	0.973	1.011	0.786	0.592	1.231	1.062	1.364	1.422	1.132	0.427	0.635	0.089
**E**	0.484	0.606	0.434	1.026	1.746	0.318	0.202	1.371	0.668	1.604	0.616	0.964
**F**	0.656	0.757	0.858	0.349	0.65	0.479	0.313	0.765	1.596	0.132	1.325	0.694
**G**	0.383	0.591	0.701	0.184	0.52	1.606	0.1	0.875	0.549	0.264	0.287	0.81
**H**	0.641	0.668	0.347	0.343	0.067	0.825	0.378	0.39	1.304	0.168	0.886	1.136

mouse Fc, OD450

	1	2	3	4	5	6	7	8	9	10	11	12
**A**	0.049	0.047	0.046	0.046	0.052	0.047	0.048	0.049	0.045	0.047	0.063	0.056
**B**	0.051	0.054	0.051	0.051	0.054	0.06	0.054	0.051	0.05	0.064	0.057	0.055
**C**	0.047	0.046	0.047	0.056	0.051	0.062	0.048	0.047	0.053	0.05	0.062	0.047
**D**	0.119	0.056	0.059	0.056	0.061	0.06	0.06	0.055	0.057	0.059	0.056	0.056
**E**	0.053	0.051	0.057	0.094	0.056	0.06	0.072	0.084	0.05	0.051	0.057	0.059
**F**	0.072	0.064	0.067	0.062	0.055	0.066	0.073	0.083	0.071	0.071	0.077	0.064
**G**	0.071	0.055	0.061	0.06	0.056	0.054	0.058	0.05	0.049	0.05	0.113	0.048
**H**	0.048	0.051	0.047	0.05	0.055	0.058	0.059	0.068	0.051	0.052	0.08	0.08

SARS-COV RBD, OD450

	1	2	3	4	5	6	7	8	9	10	11	12
**A**	0.046	0.046	0.046	0.046	0.046	0.049	0.047	0.046	0.094	0.111	0.086	0.053
**B**	0.049	0.049	0.228	0.05	0.271	0.057	0.052	0.17	0.05	0.67	0.049	0.052
**C**	0.052	0.051	0.049	0.052	0.046	0.048	0.045	0.048	0.055	0.1	0.053	0.049
**D**	0.055	0.05	0.058	0.072	0.053	0.052	0.051	0.052	0.113	0.053	0.049	0.055
**E**	0.049	0.049	0.06	0.051	0.057	0.049	0.051	0.059	0.05	0.086	0.057	0.051
**F**	0.054	0.052	0.055	0.052	0.055	0.056	0.053	0.056	0.062	0.055	0.092	0.051
**G**	0.049	0.047	0.054	0.048	0.049	0.051	0.048	0.049	0.048	0.056	0.06	0.051
**H**	0.044	0.046	0.044	0.048	0.046	0.285	0.053	0.049	0.589	0.076	0.06	0.068

-continued

PLATE #5

SARS-COV-2 RBD, OD450

	1	2	3	4	5	6	7	8	9	10	11	12
**A**	0.186	0.183	0.142	0.182	0.243	0.049	0.305	0.055	0.098	0.078	0.102	0.058
**B**	0.107	0.371	0.092	0.282	0.14	0.062	0.071	0.059	0.172	0.057	0.126	0.077
**C**	0.127	0.382	0.428	0.277	0.216	0.127	0.286	0.221	0.24	0.101	0.203	0.207
**D**	0.906	0.108	1.547	0.256	1.417	0.303	0.097	0.214	1.368	0.428	0.825	0.052
**E**	0.475	0.581	0.709	1.432	0.187	0.362	0.408	0.859	0.446	1.036	0.293	0.05
**F**	0.391	1.52	0.912	0.137	0.614	0.255	0.155	**1.385**	0.519	0.654	0.264	0.069
**G**	1.424	1.337	1.494	1.429	0.964	0.084	0.362	0.95	0.086	0.11	0.065	0.198
**H**	0.041	0.164	0.66	0.737	0.403	0.246	0.157	0.171	0.079	0.273	0.096	0.067

mouse Fc, OD450

	1	2	3	4	5	6	7	8	9	10	11	12
**A**	0.048	0.047	0.056	0.051	0.056	0.047	0.052	0.053	0.178	0.058	0.049	0.049
**B**	0.051	0.059	0.054	0.055	0.05	0.05	0.05	0.052	0.054	0.064	0.058	0.069
**C**	0.053	0.052	0.053	0.053	0.063	0.057	0.054	0.049	0.122	0.05	0.053	0.058
**D**	0.058	0.057	0.06	0.062	0.063	0.06	0.066	0.071	0.061	0.062	0.062	0.061
**E**	0.051	0.054	0.067	0.053	0.055	0.062	0.053	0.059	0.058	0.053	0.06	0.05
**F**	0.052	0.056	0.059	0.063	0.061	0.057	0.058	0.061	0.069	0.076	0.064	0.059
**G**	0.049	0.051	0.059	0.056	0.049	0.047	0.054	0.047	0.048	0.052	0.048	0.048
**H**	0.048	0.051	0.052	0.077	0.063	0.082	0.071	0.043	0.042	0.049	0.041	0.044

SARS-COV RBD, OD450

	1	2	3	4	5	6	7	8	9	10	11	12
**A**	0.071	0.077	0.075	0.076	0.081	0.071	1.095	0.074	0.806	0.069	0.071	0.067
**B**	0.075	0.243	0.074	1.071	0.076	0.080	0.077	0.073	0.071	0.072	0.072	0.070
**C**	0.072	0.073	0.073	0.074	0.348	0.072	0.787	0.078	0.435	0.069	0.074	0.072
**D**	0.606	0.078	0.076	0.077	0.074	0.085	0.079	0.074	0.083	0.070	0.073	0.073
**E**	0.073	0.075	0.073	0.079	0.075	0.075	0.077	0.079	0.072	0.072	0.074	0.072
**F**	0.071	0.077	0.382	0.074	0.072	0.464	0.074	1.036	0.073	0.070	0.121	0.066
**G**	0.076	0.077	0.072	0.586	0.075	0.074	0.079	0.071	0.069	0.065	0.069	0.067
**H**	0.078	0.079	0.079	0.083	0.076	0.078	0.077	0.073	0.074	0.071	0.075	0.074

```
                                                                  CDR1                  CDR2
                                1                                                                65
           Designed sdAb scaffold: QVQLVESGGGLVQPGGSLRLSCAASG XXXXXXXX GWFRQAPGKGLEAVAA XXXXXXXX YYADSVK
Llama VHH (GenBank: AAL58847.1):   EVQLLESGGGLVQPGGSLRLSCAASG FTFSSYAV SWVRQAPGKGLEWVSA ISGSGGST YYADSVK
 Human VH (GenBank: AAD30396.1):   EVQLQASGGGLVQAGGSLRLSCSASV RTFSIYAM GWFRQAPGKEREFVAG INRSGDVT KYADFVK

                                                                      CDR3
                                66                                                  125
           Designed sdAb scaffold: GRFTISRDNSKNTLYLQMNSLRAEDTAVYYC XXXXXXXXXXXXXXXXXX WGQGTQVTVSS
Llama VHH (GenBank: AAL58847.1):   GRFTISRDNAKNSLYLQMNSLRAEDTALYYC AKDNYDFWSG----TFDY WGQGTLVTVSS
 Human VH (GenBank: AAD30396.1):   GRFSISRDNAKNMVYLQMNSLKPEDTALYYC AATWAYDTVGALTSGYNF WGQGTQVTVSS
```

Supplementary Fig. 1. Amino acid sequences and alignment from designed sdAb scaffold in this study, llama VHH and human heavy chain VH. Three CDR regions are labeled.

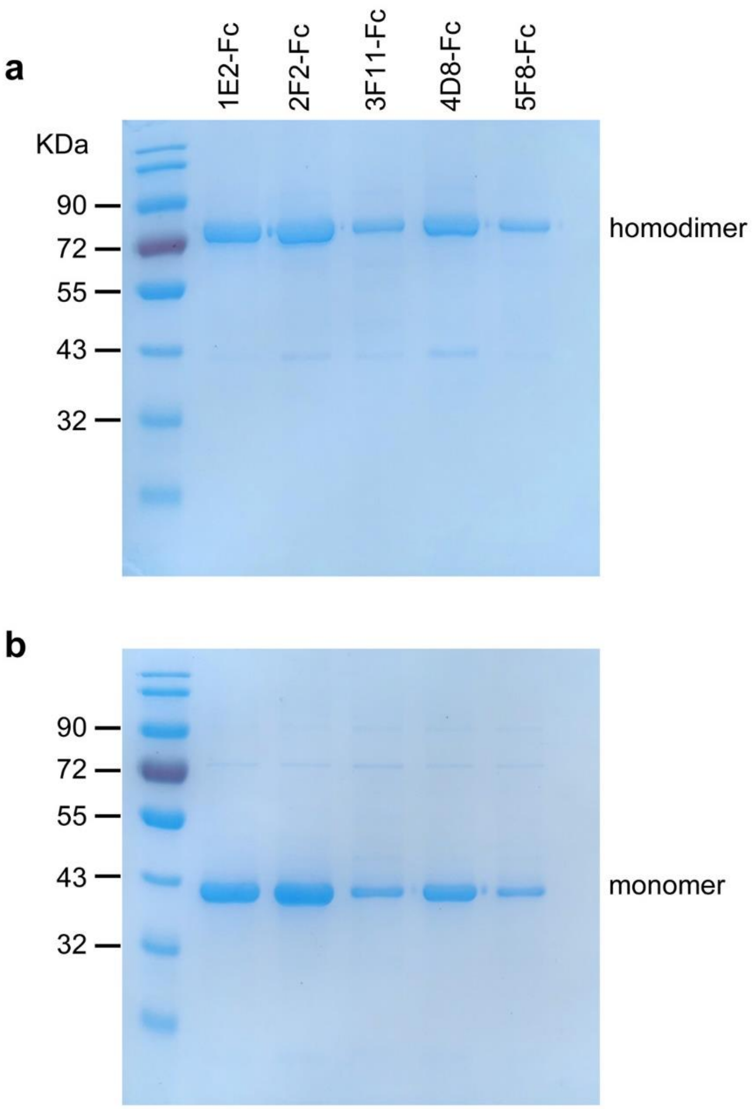

Supplementary Fig. 2. SDS-PAGE separation and Coomassie blue staining of affinity purified Fc-fusion sdAbs in non-reducing (**a**) or reducing (**b**) conditions.

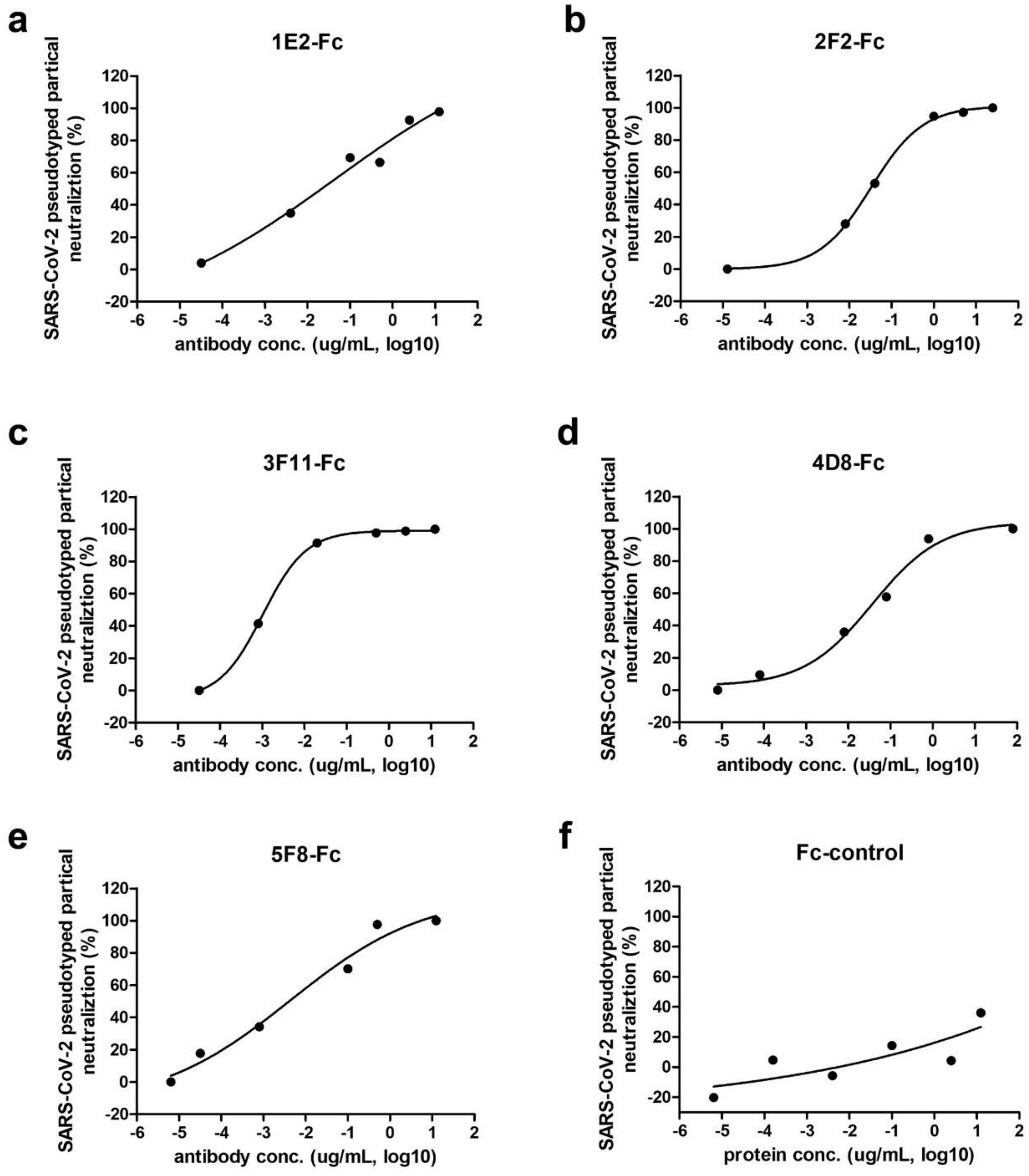

Supplementary Fig. 3. Neutralization of human IgG1 Fc-fused 1E2 (a), 2F2 (b), 3F11 (c), 4D8 (d), 5F8 (e) or Fc only (f) as control against SARS-CoV-2pp. SARS-CoV-2pp was pre-incubated with serially diluted fusion proteins before inoculation of human ACE2 transfected 293T cells. At 48 h post infection, luciferase activities were measured, and percent neutralization was calculated. The experiments were performed independently at least twice and similar results were obtained. One representative data of one experiment were shown and data were average values of two replicates.

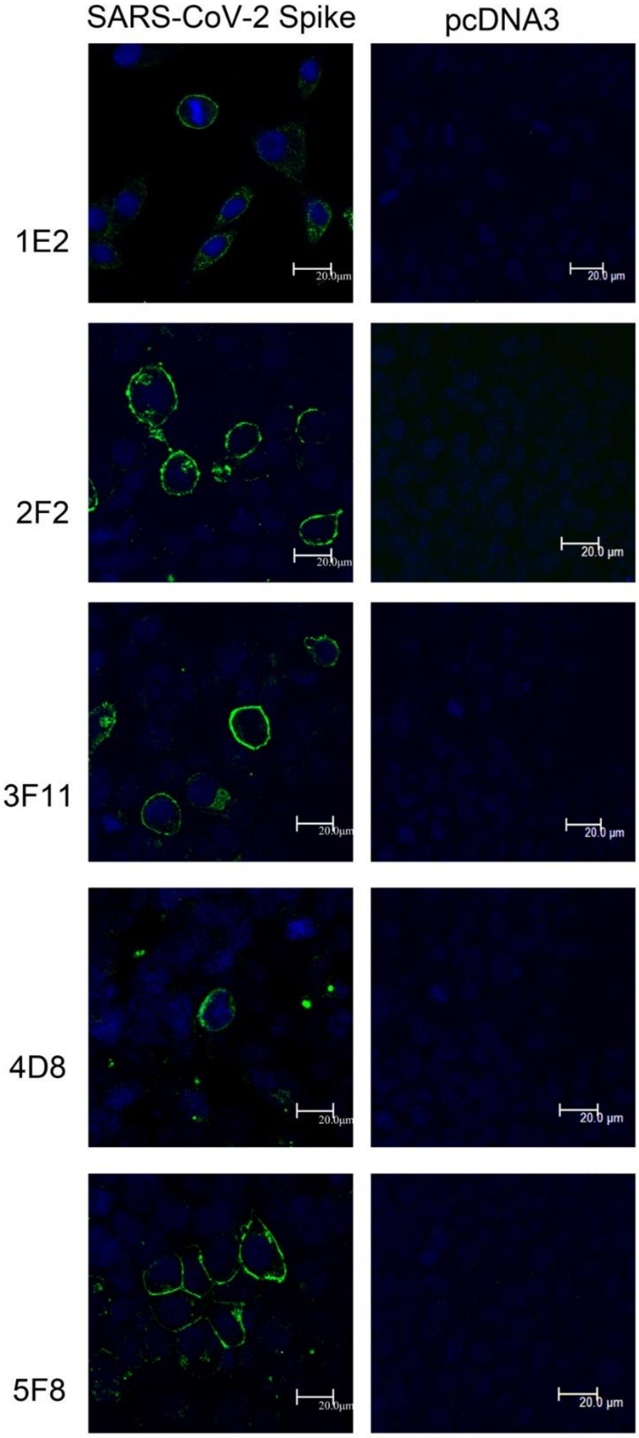

Supplementary Fig. 4. Staining of the transfected SARS-CoV-2 S protein in 293T cells with the sdAbs identified in this study. Overexpressed SARS-CoV-2 S protein was clearly localized on 293T cell plasma membrane. Scale bar, 20 μm.

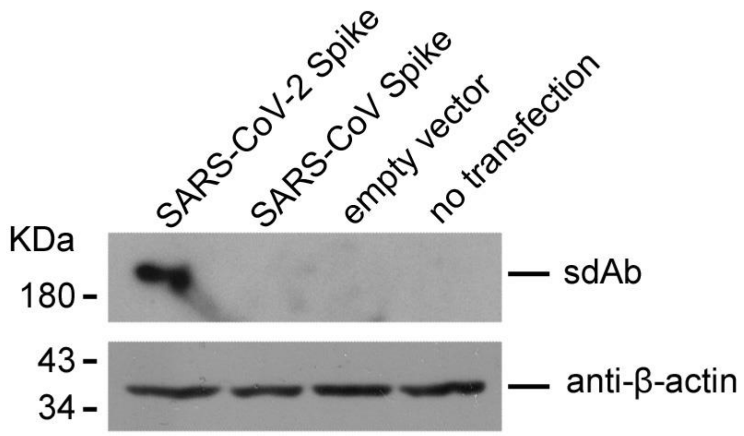

Supplementary Fig. 5. Western blot analysis shows specific recognition of SARS-CoV-2 S protein by the sdAb 2H9.
